# Exosomes as Future Therapeutic Tools and Targets for Corneal Diseases

**DOI:** 10.3390/cells14130959

**Published:** 2025-06-23

**Authors:** Joshua Gamez, Daxian Zha, Shaghaiegh M. Ebrahimi, Seok White, Alexander V. Ljubimov, Mehrnoosh Saghizadeh

**Affiliations:** 1 Biomedical Sciences, Cedars-Sinai Medical Center, Los Angeles, CA 90048, USA; joshua.gamez730@gmail.com (J.G.); daxian.zha@cshs.org (D.Z.); shaghaiegh.ebrahimi@cshs.org (S.M.E.); seokwhite@gmail.com (S.W.); ljubimov@csmc.edu (A.V.L.); 2 Board of Governors Regenerative Medicine Institute, Cedars-Sinai Medical Center, Los Angeles, CA 90048, USA; 3 David Geffen School of Medicine, University of California Los Angeles, Los Angeles, CA 90095, USA

**Keywords:** exosomes, cornea, miRNAs, biomarkers, therapeutics targets, therapeutic tools, crosstalk, cell–cell communication, exosome blockers

## Abstract

The therapeutic potential of exosomes (Exos), a subpopulation of extracellular vesicles (EVs) secreted by various cell types, has been broadly emphasized. Exos are endosome-derived membrane-bound vesicles 50–150 nm in size. Exos can be general or cell type-specific. Their contents enable them to function as multi-signaling and vectorized vehicles. Exos are important for maintaining cellular homeostasis. They are released into extracellular spaces, leading to uptake by neighboring or distant cells and delivering their contents to modulate cell signaling. Exos influence tissue responses to injury, infection, and disease by fusion with the target cells and transferring their cargo, including cytokines, growth and angiogenic factors, signaling molecules, lipids, DNA, mRNAs, and non-coding RNAs. They are implicated in various physiological and pathological conditions, including ocular surface events, such as corneal scarring, wound healing, and inflammation. Their biocompatibility, stability, low immunogenicity, and easy detectability in bodily fluids (blood, tears, saliva, and urine) make them promising tools for diagnosing and treating ocular diseases. The potential to engineer specific Exo cargos makes them outstanding therapeutic delivery vehicles. The objective of this review is to provide novel insights into the functions of Exo cargos and their applications as biomarkers and therapeutics, or targets in the cornea.

## 1. Introduction

The homeostasis of human corneal epithelial, stromal, and endothelial cells represents a dynamic and intricate process that is essential for preserving corneal transparency and ensuring optimal visual function. The cornea, a tissue responsive to constant exposure to internal and external stimuli, requires adaptive strategies to remain transparent and perform the major refractive function of the eye [1]. Corneal cells regulate their niche environment through interactions with the extracellular matrix (ECM) and intercellular crosstalk within and/or among different corneal layers to maintain their homeostasis or contribute to pathophysiology [2]. Corneal epithelial function mainly depends on the health of the microenvironment composed of stem cells residing in the corneoscleral region of the limbus [3,4]. The intercellular communication of limbal niche cells, for example, limbal epithelial stem cells (LESCs), the source of corneal epithelium, and limbal stromal stem cells (LSSCs), the source of keratocytes, is a key factor in their maintenance and activation [5,6]. Globally, visual impairment remains a significant public health issue, affecting approximately 53 million individuals who experience blindness and 295 million with moderate-to-severe visual impairment as of 2020 [7]. Disorders impacting any layer of corneal cells—such as limbal stem cell deficiency, corneal dystrophies, bullous keratopathy, or injuries resulting in fibrosis—can lead to corneal opacity and subsequent vision disturbances [8]. The human cornea is susceptible to damage from systemic illnesses, such as viral and bacterial infections, genetic conditions, congenital disorders, and diabetes [9,10]. It has been shown that the human cornea is highly susceptible to oxidative stress due to its direct exposure to ultraviolet radiation, elevated metabolic activity, and high oxygen tension. These factors collectively contribute to oxidative injury, playing critical roles in the development and pathogenesis of multiple corneal diseases, including keratoconus, pterygium, and chemical and traumatic injuries, as well as various inflammatory, degenerative, and metabolic disorders [11].

A growing number of studies have investigated extracellular vesicle (EV)-mediated cell–cell communication and its implications in disease progression [12,13,14,15,16]. EVs are considered one of the major transport mechanisms in corneal and limbal niche cells [17,18]. EVs are nano-sized, membrane-bound vesicles ranging from 50 to 1000 nm in diameter, encompassing subtypes such as exosomes (Exos), microvesicles, and ectosome vesicles. These vesicles, which differ in their subcellular origin, mode of release, and size, are secreted by most cell types mediating physiological intercellular communication [12,19,20,21]. According to the latest MISEV (Minimal Information for Studies of Extracellular Vesicles) guidelines, Exos are a subtype of small EV, with intraluminal vesicles of endosomes typically measuring less than 200 nm in diameter [22]. They are composed of a variety of proteins, metabolites, and nucleic acids that reflect the cells and tissues of origin [12,20,22]. Exos are formed within endosomes, which subsequently fuse with the plasma membrane and are released as multivesicular bodies into extracellular spaces, facilitating both extracellular and intercellular communication [13]. Exos are secreted by diverse cell types and present in biological fluids, including serum, milk, cerebrospinal fluid, urine, saliva, and ocular fluids (tears and vitreous and aqueous humor) [23]. The transfer of biomolecules, such as proteins and RNA, between cells is facilitated by Exos, which play a crucial role in enabling intercellular communication [12,24]. Evidence of Exos’ involvement has been found in immune regulation, inflammatory responses, and neovascularization [25,26,27]. The cellular cross-effects of Exos depend on the biological properties of their cargos, including proteins (e.g., growth factors, cytokines, and hormones) and miRNAs (e.g., immunoregulatory, anti-/pro-apoptotic, and survival-related miRNAs), as well as mRNAs, DNA, lipids, small molecules, and metabolites [13,28,29,30,31]. Investigating the molecular cargo carried by Exos and their impact on corneal physiology could significantly enhance our understanding of disease pathways, facilitate biomarker discovery, and enable the creation of novel therapeutic approaches.

In this review article, we present a brief overview of Exos and their biogenesis, followed by the modulatory functions of exosomal cargos in diseased corneas and their possible role as promising biomarkers. We present a comprehensive review of current studies on Exos, including natural and engineered exosomal cargos, highlighting their roles in the progression, diagnosis, and potential treatment of corneal diseases.

## 2. Overview of Exosomes

Exos are small membrane-bound microvesicles originating from endosomes, which have garnered considerable attention in recent years. Initially observed in the extracellular environment during the early to mid-1980s, they were first identified in maturing mammalian reticulocytes by the research groups of Stahl and Johnstone in 1983 [32,33]. Subsequent studies revealed that Exos function as natural carriers facilitating the transfer of nucleic acids, proteins, and lipids between donor and recipient cells through autocrine, paracrine, and endocrine pathways [24,34,35]. The specific cargo and composition of Exos can vary depending on the originating cell type and prevailing physiological or pathological conditions. As mediators of intercellular communication, Exos play a pivotal role in cellular signaling by delivering their molecular cargos to target cells [36,37]. Extensive research has advanced our understanding of Exos’ structure. Electron microscopy studies have provided clear evidence of Exos’ morphology, their distinct cup-shaped or spherical structures and confirmed their externalization through the fusion of multivesicular bodies with the plasma membrane [38]. The biogenesis of Exos was initially demonstrated by observing the shedding of membrane-bound ectoenzymes as microvesicles [39]. Subsequently, further insights came from studies documenting the externalization of transferrin receptors within vesicular structures derived from reticulocytes [24,40].

**Figure 1:**
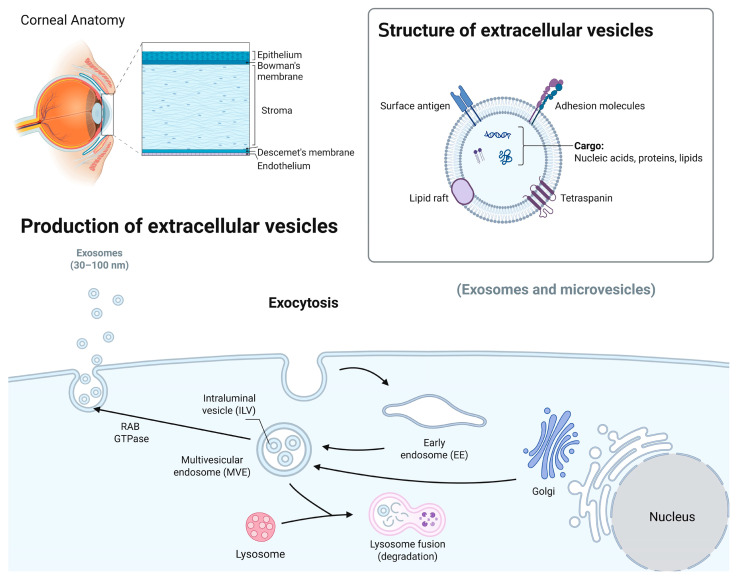
A schematic overview of the corneal structures and biogenesis of Exos. The human cornea, the transparent outer part of the eye, consists of several distinct layers, including the epithelium, Bowman’s membrane, stroma, and Descemet’s membrane and endothelium. Exos carry nucleic acids, proteins, and lipids and are involved in intercellular communication. Their biogenesis involves the formation of early endosomes, leading to the formation of intraluminal vesicles (ILVs) through inward membrane budding and maturation into multivesicular bodies (MVBs). The MVBs can either be degraded by lysosomes or released as Exos outside the cell using Rab GTPase and SNARE complexes (image created in BioRender).

Exos’ biogenesis is a complex process, involving several important stages, such as cargo sorting, formation and maturation of multivesicular bodies (MVBs), their transport, and fusion with the plasma membrane to release Exos [41]. This intricate mechanism guarantees the selective packaging of specific cargos into Exos and their consequent release into the extracellular milieu [13]. During Exos’ biogenesis, selective sorting and packaging of various molecules occur through specific mechanisms, including endosomal sorting complexes required for transport (ESCRT) machinery, tetraspanins, and lipid-dependent sorting (Figure 1) [42,43]. These sorting mechanisms ensure the inclusion and exclusion of specific cargo molecules. MVBs play a central role in Exos’ biogenesis and are formed through the inward budding of the endosomal membrane, generating intraluminal vesicles (ILVs) within the MVB lumen. Exos’ biogenesis involves the ESCRT system, comprising ESCRT-0, ESCRT-I, ESCRT-II, and ESCRT-III, which are complexes responsible for the selective sorting and packaging of various molecules into Exos [41,44]. Other proteins, such as ALG-2-interacting protein X (ALIX) and syntenin, also contribute to the maturation and formation of MVBs [44,45]. Following their formation, MVBs can undergo different intracellular trafficking routes, either fusing with lysosomes for degradation or with the plasma membrane, leading to the release of ILVs as Exos [46].

The regulation of MVB trafficking involves various molecules, such as Rab GTPases [47], Soluble NSF Attachment Protein Receptor (SNARE) proteins [48], and lipids [41]. The final step in the biogenesis of Exos involves the fusion of MVBs with the plasma membrane, leading to the release of ILVs as Exos into the extracellular space. This membrane fusion process is facilitated by the interactions of ESCRT proteins, SNARE proteins, and calcium ions. Upon fusion, ILVs are released as functional Exos [41].

## 3. Exosomal Cargos

In recent years, researchers have made significant progress in understanding Exos’ role in biological processes and the mechanisms of cargo sorting and packaging [49]. Exos are capable of transporting a variety of biomolecules, with their content varying based on several factors [50,51]. Exosomal cargos can influence the behavior of the recipient cells. This is supported by a study by Ramos et al. demonstrating that human conjunctival epithelial cells treated with corneal epithelial-derived EVs expressed the corneal-associated keratin (KRT) markers, KRT 3 and KRT12. Simultaneously, human corneal epithelial cells treated with conjunctival-derived EVs expressed the conjunctiva-associated markers KRT7 and KRT13 [52]. Another study found that small EVs (sEVs) derived from limbal epithelial stem/progenitor cells (LEPCs) contained proteins essential to epithelial cells and their development, such as keratins, S100 proteins, serpins, and desmosomal complex proteins. Additionally, many of these proteins were exclusive to sEVs derived from LEPCs and not from other corneal cell types. Using gene ontology analysis, it was confirmed that the proteins found in these sEVs were involved in critical epithelial cell pathways, such as wound healing, cell–substrate adhesion, ECM receptor interaction, and peptidase activity [53]. These findings suggest that the differences in the cargo carried by the EVs can trigger cellular trans-differentiation [54]. Exos serve as delivery vehicles for proteins, nucleic acids, lipids, and potential small drug molecules [41]. Exosomal nucleic acids cargo includes both mRNAs and non-coding RNAs (ncRNAs), such as miRNAs, long ncRNAs (lncRNAs), circular RNAs (circRNAs), ribosomal RNAs (rRNAs), transfer RNAs (tRNAs), small nucleolar RNAs (snoRNAs), small nuclear RNAs (snRNAs), and piwi-interacting RNAs (piRNAs). In addition, exosomal proteins may include growth and angiogenesis factors, cytokines, chemokines, and other functional molecules. Therefore, Exos can initiate distinct functional responses in the target cells by transporting these nucleic acids and proteins from parent cells to recipient cells [55,56,57,58,59].

Further, mRNAs and enriched ncRNAs within Exos are more stable than their circulating counterparts because of protection by the lipid bilayer [60,61]. These exosomal ncRNAs have been found to modulate multiple cellular processes by regulating gene expression [62]. Enriched lipids have been shown to play an important role in Exos’ structure and function as well. Such lipids include cholesterol, phosphatidylserine, ceramides, sphingolipids, and glycerolipids [63]. The lipid composition of Exos has been found to significantly impact their biogenesis, stability, cargo sorting, and interactions with target cells [63]. Although the mechanism of the selective packaging of Exos is still not fully understood, it has been shown that Exos from different sources or the same cells under different conditions contain unique cargo profiles [64]. These exosomal cargos reflect the content and existing status of the originating cells. Therefore, Exos may serve as potential diagnostic or prognostic biomarkers and a therapeutic tool.

## 4. Exosome Isolation and Characterization

Certain techniques for processing and isolating Exos can generate high purity and yield that are optimal for diagnostic and therapeutic applications. The quality of the isolated Exos significantly depends on multiple factors, including composition, separation method, and sample type. The most common techniques for Exos’ isolation are ultracentrifugation, ultrafiltration, immunoaffinity capture precipitation, and size-exclusion chromatography, which are based on their density and size, as well as their solubility and specific binding using antibodies [65,66,67,68,69,70]. Ultracentrifugation is considered the gold standard for Exos’ isolation because it can effectively isolate microscopic particles like Exos by separating components based on density [70]. Differential ultracentrifugation is often utilized and involves multiple rounds of centrifugation to separate Exos from larger vesicles and cell debris [71]. These traditional methods can be used to obtain high-purity samples; however, the high recovery yield is not guaranteed. For example, differential ultracentrifugation requires several rounds of centrifugation and repeated removal and transfer of the supernatant into new sample tubes, resulting in Exos’ damage and loss, leading to low yield [72]. To offset these effects, larger amounts are used to obtain the desired yield. Researchers have often combined different methods, such as ultracentrifugation and ultrafiltration, to improve both purity and yield; however, this can be costly and time-consuming [73,74]. Another caveat of Exos’ isolation methods is the lack of high-quality standards. These traditional isolation methods are used widely in research; however, they have limitations in clinical applications, which hinder the progression of the field. Currently, there are no FDA-approved Exos products to be used for disease diagnosis or treatment [75]. The major role of Exos in intercellular communication and their presence in body fluids make them an important predictor of disease progression and a potential biomarker for diagnosis [76,77]. Therefore, it is necessary to introduce more efficient techniques to purify and yield Exos with a high standard of quality.

In recent years, new isolation methods have been developed to improve the yield and purity of Exos while maintaining their bioactivity [66]. Microfluidic technology has been in the spotlight because it provides high resolution and sensitivity for Exos’ separation and analysis [78]. This technology has great potential in clinical research and can provide point-of-care testing for patients, which the EV field is currently lacking. However, microfluidics technology for Exos’ isolation is still under development, can be expensive, and requires a lengthy manufacturing process [79]. It has been shown that isolation methods can introduce variations in Exos’ purity, concentration, and size [80]. In one study, an RNA-seq analysis of Exos isolated by two different methods, ultracentrifugation and ExoQuick or Total Exosome Isolation Reagent, showed different small RNA profiles. It was concluded that different isolation methods may introduce variations in the concentration, purity, and size of the exosomal RNA. The Exos across all different samples contained 588 common and about 200 unique miRNAs for each isolation method [80]. Gardiner et al. reported a similar result, noting that proteomic or flow cytometry-based analyses of Exos are often skewed by contaminating co-isolated plasma proteins. The amount of contamination depends on the isolation method used, which includes ultracentrifugation, size exclusion chromatography, filtration, precipitation, and magnetic bead capture [81]. Recently, Chen et al. introduced a novel approach for Exos’ detection termed the ultrafast-isolation system (EXODUS), an automated, label-free purification technique with benefits in terms of speed, purity, and output. This method employs negative pressure oscillations combined with double-coupled resonators to produce transverse waves at dual frequencies on nanoporous membranes. The resulting membrane vibrations effectively reduce fouling, preventing clogging issues and enabling rapid, efficient purification of Exos [82]. Although EXODUS seems a promising method ensuring better purity and yield of isolated Exos, further research is still needed to develop and optimize it for higher yield and purity while maintaining Exos bioactivities for clinical applications.

Proteins also play an important role in Exos in addition to miRNA and other nucleic acids. Exos isolated by different techniques reveal varying levels of their protein contents [83]. The identified variable proteins, such as tetraspanins that comprise CD9, CD63, and CD81, are the most used biomarkers to identify Exos [84]. The differences in protein content may be due to different EV subpopulations in each isolated sample [77]. The process of Exos’ isolation is often overlooked in EV research, which presents a major challenge in the field. The lack of uniformity among Exos’ isolation methodology can potentially invalidate findings. As mentioned previously, the purity of Exos and their cargos is highly dependent on the isolation technique, which can affect outcomes and subsequent analyses. Despite these challenges, exosomal cargo analyses emerge as a promising diagnostic tool in clinical applications because of their ability to facilitate cellular communication and influence target cell function. These limitations in the EV field can be addressed with a thorough evaluation of the results to validate the important role of Exos’ cargos as messengers between donor and recipient cells and their influence on cell behavior.

EV characterization is essential to confirm their presence, estimate abundance, and evaluate potential contaminations from non-vesicle components. Due to their small size, varied composition, and absence of a universal EV identification marker, no single method can completely characterize EVs. The MISEV2023 guidelines provide a comprehensive framework to ensure standardization with emphasis on source-specific validation, transparency, and methodological rigor [22]. Commonly used analytical and quantitative techniques for EV characteristics include nanoparticle tracking analysis (NTA; Nanosight and ZetaView), Spectradyne, Exoview, nanoflow cytometry (NanoFCM or CytoFLEX nano) for single EVs phenotyping, resistive pulse sensing (RPS), multi-angle light scattering, and dynamic light scattering (DLS). all of which are employed for quantifying particle size and concentration, typically under the assumption that EVs are spherical [22]. For protein content, total protein in an EV preparation can be estimated using colorimetric or fluorometric assays, size exclusion chromatography, SDS-PAGE (Sodium Dodecyl Sulfate Polyacrylamide Gel Electrophoresis) with global protein staining, or absorbance measurements, each varying in sensitivity and specificity [22]. Morphological characterization is best achieved using high-resolution imaging techniques such as scanning electron microscopy (SEM), transmission electron microscopy (TEM), cryo-electron microscopy (cryo-EM), and atomic force microscopy (AFM) [22]. These complementary methods provide insights into EV size distribution, purity, structural integrity, and molecular composition, and their combined application is critical for robust, reproducible EV analysis.

## 5. Dual Role of Exosomes in Cell Communication and Therapy as Tools and Targets

In addition to the important role that Exos play in intercellular communication and transporting a diverse range of biological molecules between cells, they are well recognized as a tool for delivering therapeutic products [85]. Exos have a broad range of influence on various physiological and pathological processes depending on their contents [37]. Their cargo also depends on the originating parent cells and the condition of the microenvironment [86]. Exos derived from healthy cells or therapeutically active cells, such as mesenchymal stem cells (MSC), can exert the positive effects of facilitating tissue restoration and homeostasis maintenance through their protective cargo transport [87]. In such scenarios, boosting Exos’ secretion or Exos’ engineering with bioactive components is a promising therapeutic approach to amplify beneficial effects, especially in regenerative medicine. While Exos have beneficial effects on recipient cells, their implication in disease states, such as cancer, can promote metastasis and angiogenesis [88], and in neurodegenerative diseases, such as Alzheimer’s, can carry misfolded beta-amyloid protein, potentially spreading pathology [89]. In such contexts that Exos can exert deleterious effects, inhibition of their formation or release becomes a promising therapeutic strategy.

### 5.1. Exosomes as Treatment Tools and Biomarkers in Corneal Diseases

Exos have emerged as highly promising diagnostic and therapeutic tools for various ocular diseases, including age-related macular degeneration (AMD), diabetic retinopathy (DR), glaucoma, dry eye, diabetic keratopathy, and corneal fibrosis [90,91,92,93,94]. Their unique ability to transport a diverse array of bioactive molecules—such as non-coding RNAs, mRNAs, DNA, proteins, and lipids—to both neighboring and distant recipient cells has positioned them as a leading platform for next-generation delivery systems in disease management.

Exos have demonstrated significant potential in corneal therapy by promoting healing, reducing inflammation, and modulating cellular behavior. The functional impact of Exos is largely determined by their cargo composition. For instance, our lab has demonstrated notable differences in the small RNA cargo profiles of Exos derived from human limbal stromal cells (LSCs) of diabetic patients compared to those from non-diabetic individuals [18]. The altered miRNA composition in diabetic vs. non-diabetic LSC-derived Exos was linked to changes in cellular behavior, potentially contributing to impaired corneal epithelial wound healing and regeneration, as well as the downregulation of limbal epithelial stem cell markers in diabetic conditions. It has also been shown that Exos derived from human corneal mesenchymal stromal cells significantly accelerated epithelial wound healing in experimental models and attributed to the regenerative properties of their cargo, which enhanced cell proliferation and migration, critical processes in wound repair [95]. Further, our lab has revealed miRNA and proteome profile differences in diabetic vs. non-diabetic LEC-derived Exos’ cargos, including proteins involved in Exos’ biogenesis and packaging that may affect Exos’ production and corneal function [96]. Moreover, non-diabetic-LEC Exos had a more pronounced effect on LSC wound healing, proliferation, and stem cell marker expression than diabetic-LEC Exos [18]. Research has also shown the multifaceted role of Exos released by corneal epithelial cells by facilitating intercellular communication between keratocytes, epithelial, and vascular endothelial cells, which is important in promoting tissue repair and regulating neovascularization [17]. Exos from human corneal cells have also been shown to activate key signaling pathways in recipient cells, such as STAT, GSK-3β, β-catenin, and p38 [97].

Beyond wound healing, Exos offer anti-inflammatory and antifibrotic benefits, making them particularly valuable for chronic corneal conditions. For example, Wu et al. encapsulated lutein, a potent antioxidant, in milk-derived Exos to improve its stability and bioavailability. In a dry eye disease (DED) mouse model, this approach reduced inflammation and repaired epithelial damage, showcasing the combined benefits of lutein’s antioxidant properties and the biocompatibility of Exos [98]. Similarly, EVs derived from human amniotic epithelial cells demonstrated efficacy in reducing inflammation and promoting repair in a DED model [99]. Bone marrow MSC-derived Exos were recently studied as potential drug carriers. The results showed that Exos carrying miR-29b-3p significantly inhibited corneal fibrosis and inflammation associated with corneal injury [100]. Collectively, these studies emphasize the versatility and therapeutic potential of Exo-based approaches in corneal therapy. By modulating cellular behavior, promoting intercellular communication, and delivering targeted bioactive molecules, Exos hold promise for addressing a variety of corneal conditions, including wound healing deficits, chronic inflammation, fibrosis, Neuro-Ophthalmic Disorders, and dry eye disease [99,100,101,102,103,104]. They also showcase the significant potential of engineered Exos for therapeutic purposes.

**Figure 2:**
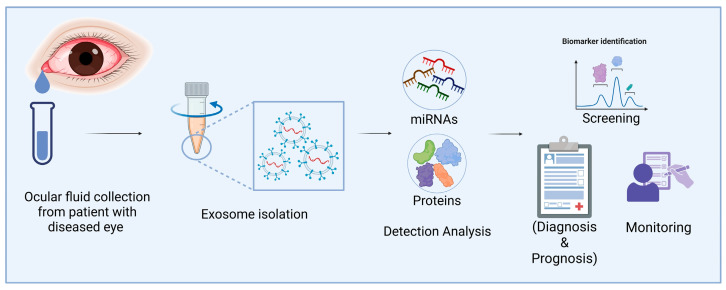
Applications of eye fluid-derived exosomal miRNA and proteins as diagnostic biomarkers of corneal diseases. Exos from eye fluids can be analyzed for omics data. These Exos may be useful for diagnosing corneal diseases after marker validation (Image created in BioRender).

Compared to other molecular agents, such as viruses, EVs have been found to exhibit lower immunogenicity and greater stability in biological fluids, making them promising carriers for potential biomarkers in the diagnosis of ocular diseases [105]. Recent studies have also investigated biomarkers derived from EVs isolated from ocular fluids in disease models, such as dry eye disease (DED) [106], Sjögren’s syndrome (SS) [107], diabetic retinopathy [108], and keratoconus [109]. Figure 2 presents an example workflow illustrating the collection and analysis of ocular fluids for omics-based profiling. Following appropriate biomarker validation, these EVs may serve as valuable tools for the diagnosis of corneal diseases.

#### 5.1.1. Exosomal miRNAs and Their Therapeutic Potential

Exosomal miRNAs are emerging as pivotal therapeutic agents due to their critical roles in gene regulation and intercellular communications. These small, non-coding RNAs are selectively packaged into Exos and released by donor cells, enabling the targeted modulation of gene expression in recipient cells. Their remarkable stability, conservation across species, and functional diversity underscore their importance in both physiological and pathological processes [37,110]. The regulatory pathways mediated by exosomal miRNAs include their selective packaging into Exos, release from donor cells, uptake by recipient cells, and subsequent modulation of gene expression [111]. Upon release into the extracellular environment, Exos are internalized by recipient cells via mechanisms such as endocytosis, phagocytosis, or membrane fusion [112]. Once inside, exosomal miRNAs interact with the RNA-induced silencing complex (RISC) and bind target mRNAs through sequence complementarity, resulting in translation inhibition or mRNA degradation [113,114]. This finely tuned regulation underpins their role in diverse diseases, including cancer [61], cardiovascular conditions [29], and central nervous system disorders [115], with implications for angiogenesis, metastasis, and immune modulation [116,117].

Given their abundance, stability in body fluids, and ease of detection, exosomal miRNAs have been proposed as biomarkers for disease diagnosis and therapeutic targeting. In ophthalmology, they have shown diagnostic potential for conditions such as corneal infections, neovascularization, and diabetes [118,119]. Dysregulated miRNAs, such as those implicated in Fuchs’ endothelial corneal dystrophy (FECD) [120] and corneal diabetes [121], highlight their role in disease pathogenesis and progression. For example, aberrant miRNA expression has been linked to impaired wound healing, macrophage migration, and ECM remodeling in conditions such as corneal fungal keratitis and FECD [122,123].

Exosomal miRNAs hold promise not only as diagnostic biomarkers but also as therapeutic agents due to their ability to cross biological barriers and deliver regulatory molecules to target tissues [124]. Recent studies showed that miR-146a and miR-10b are differentially expressed in diabetic vs. healthy limbal progenitor cell-derived Exos, suggesting their role in impaired epithelial wound healing and/or stem cell maintenance/differentiation via targeting the epidermal growth factor receptor (EGFR) and Wnt signaling, respectively, in diabetic corneas [18,96,119,121]. Notably, administration of a miR-146a inhibitor improved epithelial healing in diabetic human organ-cultured corneas [119]. This approach presents a compelling therapeutic option for patients suffering from diabetes-related corneal complications. It was found that Exos enriched in miR-24-3p effectively promote corneal epithelial repair, indicating their suitability for epithelial regeneration strategies [125]. Another important example involves mouse amniotic fluid-derived MSC Exos (mAF-MSC-Exos), which facilitate the repair of corneal cryoinjury models through the delivery of the enzyme DNA methyltransferase 1 (DNMT1). This results in hypermethylation and the subsequent downregulation of miR-33, ultimately elevating Bcl6 expression. The resulting molecular changes significantly enhance corneal epithelial cell regeneration and structural restoration [126].

Further reinforcing this strategy, MSC-derived Exos enriched with specific miRNAs, notably miR-29 and miR-155, were found to upregulate antifibrotic and pro-regenerative pathways [127,128]. These miRNAs significantly reduced fibrotic markers such as TGF-β1 and α-SMA in corneal tissues, emphasizing their therapeutic efficacy in preventing corneal fibrosis and subsequent scarring [128]. Additionally, exosomal miR-19a from adipose-derived stem cells (ADSCs) has been reported to suppress fetal bovine serum-induced differentiation of rabbit corneal keratocytes into myofibroblasts by inhibiting homeodomain-interacting protein kinase 2 (HIPK2) expression. This finding further illustrates the capability of specific exosomal miRNAs to regulate cellular differentiation pathways to mitigate fibrosis [129].

Furthermore, exosomal miR-223-3p from mouse adipose-derived MSCs (mADSC-Exos) effectively mitigates ocular surface inflammation and damage associated with dry eye disease by directly targeting and suppressing F-box and WD repeat domain-containing protein 7 (Fbxw7) expression. This positions miR-223-3p as a viable therapeutic candidate with considerable potential for addressing dry eye disease-related pathologies [130]. Small EVs derived from human umbilical cord MSCs (HUMSC-sEVs) have been shown to significantly enhance corneal epithelial wound healing by delivering miR-21. MiR-21 effectively inhibits Phosphatase and Tensin homolog (PTEN) expression, thereby activating the PI3K/Akt signaling pathway, a mechanism that highlights HUMSC-sEVs as a promising therapeutic agent for promoting corneal cell survival and wound healing and providing immunomodulatory benefits [131].

These findings collectively highlight the potential of miRNA from Exos as an innovative therapeutic agent for ocular surface diseases. They also emphasize the integral role of exosomal miRNAs in facilitating intercellular communication between ocular tissue cells, maintaining homeostasis, and contributing to tissue repair. Continued exploration of exosomal miRNAs in ocular pathologies, particularly their interactions with immunoregulatory molecules, could unveil transformative therapeutic avenues. Exos’ integration into personalized medicine and as carriers for miRNA-based therapies is an area ripe for exploration, potentially revolutionizing the management of ocular diseases and beyond. Their use in regenerative medicine and immunomodulation presents a novel approach to cell-free therapies, mitigating the side effects associated with conventional treatments [23,132]. Advances in omics-based analyses of Exos derived from ocular fluids may further refine their diagnostic and predictive utility for corneal diseases [133]. However, one needs to realize that the scale-up of therapeutic Exos to produce large quantities for potential human use still presents a significant problem.

#### 5.1.2. MSC-Derived Exosome-Based Therapy

Mesenchymal stem cells (MSCs) are a type of cell that can be isolated from a variety of tissues such as the umbilical cord, bone marrow, adipose tissue, skeletal muscle, dental tissue, and cornea [21,59,95,100,131,134]. It is well established that MSCs can be used as a potential therapeutic tool for many diseases due to their anti-inflammatory, regenerative, and immune regulatory effects [134]. Studies have demonstrated that MSC-derived Exos (MSC-Exos) can be a safer and more advantageous alternative to MSC therapy [21,87,95,127,134]. MSC-Exos produce effects similar to their parental cells. Additionally, as a cell-free treatment, they are less immunogenic, more permeable, and more stable therapeutic tools [135].

Clinical trials using MSCs and MSC-Exos for the treatment of several diseases, including corneal abnormalities, have shown great promise [136,137]. A study found that treatment with MSC-Exos resulted in reduced endoplasmic reticulum (ER) stress, as demonstrated by decreased levels of proteins involved in ER stress, such as Eukaryotic Initiation Factor 2 (2EIF2), Activating Transcription Factor 4 (ATF4), and changed caspase-3 and Akt levels, leading to a decrease in apoptotic cells in vitro [138]. Similar studies have shown that corneal MSC-derived Exos increase the healing of corneal epithelial cells [95] and inhibit fibrosis after corneal injury in mice [139]. Several in vivo and clinical studies have shown the significant potential of MSCs in the treatment of dry eye syndrome as well [140,141].

Corneal wound healing is a complex process that includes differentiation, cell death, proliferation, migration, and ECM remodeling that is regulated by various biological factors. A study of proteomics data on small EVs (sEVs) derived from mesenchymal stromal cells (LMSC) and melanocytes (LM) revealed that these sEVs contain proteins that play a role in ECM remodeling, ECM receptor interaction, collagen synthesis, and the regulation of the collagen-rich ECM. This underscores the role of sEVs in intercellular communication, microenvironment sensing, and alterations in ECM dynamics. Proteomics data revealed distinct molecular cargos reflecting the sEVs’ biological roles: LM-sEVs for pigmentation, cell structure, and oxidative protection; and LMSC-sEVs for ECM composition, cell–matrix adhesion, and wound healing [142].

In recent years, the role of miRNAs in corneal wound healing has been investigated, and studies have shown their potential as novel therapeutic strategies [143,144]. Differentially expressed miRNAs identified in diabetic MSC-derived Exos vs. non-diabetic corneal MSC-derived Exos by next-generation sequencing may contribute to corneal wound healing and the diabetic disease state. It has been shown that wound healing rates in primary limbal epithelial cells and organ-cultured corneas were significantly enhanced upon treatment with non-diabetic Exos but not by diabetic Exos [18]. In another study, small EVs derived from human umbilical cord MSCs transfected with miR-21 were found to promote wound healing in human corneal epithelial cell lines [131]. Additionally, treatment of injured rat corneas with human MSCs (HUMSCs) increased healing; however, HUMSCs treated with the Exo blocker GW4869 decreased the healing effects [131]. All these results are a promising indication for the use of MSCs and their Exos in conjunction with miRNAs to promote corneal healing. Given that MSCs and their Exos can be harvested from a variety of sources and that they have shown much potential as a treatment for a variety of ocular afflictions, continued research about them will be invaluable for developing accessible and effective treatments.

#### 5.1.3. Modified-Exosome-Based Therapy

As a form of Exo-based therapy, promising studies explored ways to modify and engineer specific Exo cargo for different therapeutic applications [145]. To use modified Exos for treatment, a recent study by Wiklander et al. demonstrated that engineered EVs displaying Fc-binding domains can serve as a versatile platform for targeted cancer therapy, allowing antibody-directed delivery to achieve selective targeting and therapeutic efficacy in mouse cancer models [146]. Therefore, engineered EVs equipped with Fc-binding domains represent a flexible delivery system that can be decorated with IgG antibodies to potentially enable targeted delivery to specific ocular tissues, offering precision therapies in ocular surface diseases. Another approach is protein-based therapeutics engineered by fusing the target proteins to the cytoplasmic domain of EV-sorting proteins, such as CD63, which remain bound to the membrane [147]. To address this limitation, the EXPLORs (exosomes for protein loading via optically reversible protein–protein interactions) system was developed, introducing cleavable linker peptides between the target protein and the EV-sorting domain [148]. A recent study employed self-cleaving mini intein-based cargo release and the fusogenic protein VSV-G (viral G-protein) to enhance EV-mediated intracellular protein delivery, serving both as an endosomal escape facilitator and an EV-sorting component [149]. One may target the expression of pathological miRNAs by including antagonistic agents in Exos that would suppress disease development. For instance, Exo cargo loaded with anti-miRNA oligonucleotides could be applied by topical delivery through eye drops or local injection. To address difficulties in penetrating the human cornea due to a large molecule size and tight epithelial barrier, these delivery methods can be modified for what is more feasible. Exos that are amenable to modifications can be considered promising therapeutic candidates to suppress disease progression [17,129,150,151,152].

Recent research has shown that one of the five members of the NF-κB family, c-Rel, is preferentially expressed by immune cells and promotes the expression of inflammatory cytokines that may delay corneal wound healing. Further studies showed that modified Exos loaded with NF-κBc-Rel-specific siRNA (siRel) can significantly accelerate wound healing in both healthy and diabetic corneas [153]. Additionally, to treat corneal inflammation, Exos derived from EGF-treated M1 macrophages applied as eye drops effectively reduced inflammation in murine corneas [154]. The proteomic profiles of these Exos were mostly related to immune regulation and the inhibition of inflammation [154].

Exos can also be engineered to modify their cargo to allow improvement of their natural properties for targeted therapies. Exo engineering can be undertaken to increase the efficacy of drug delivery and gene therapy, as well as immunomodulation, by modifying the surface features and composition. Molecular engineering makes it possible to isolate Exos that are more stable, selective, and efficient [155,156]. Employing Exos derived from induced pluripotent stem cells (iPSCs) has the potential to serve as an alternative therapy to provide an efficient cell-free regenerative medicine approach toward diseases. As shown by Wang et al., iPSC-derived Exos displayed better therapeutic effects and promoted corneal epithelial wound healing in vivo compared to Exos isolated from MSCs [157]. In vitro, they also more effectively enhanced cell migration, proliferation, and cell cycle progression than MSC-derived Exos [157]. A cell-free therapeutic approach to address corneal wounds and other ocular surface diseases could involve the use of iPSC-derived Exos in eye drop form. Exos derived from iPSCs may serve as the source of therapeutic Exos that can be engineered and utilized for the treatment of various diseases, as well as future translational research.

As mentioned previously, Exo-based therapy can be delivered topically. However, drug delivery efficiency through the human cornea is a significant issue that needs to be addressed. In the context of vitreoretinal diseases, topical treatment is difficult due to slow corneal uptake and clearance. To address these concerns, high-purity milk-derived Exos were engineered by anchoring arginine-rich cationic motifs via PEG2000 lipid insertion on their surface [158]. These modified Exos use electrostatic interactions with anionic glycosaminoglycan and water content to enhance their transport rate and retention. Cationic-motif-modified Exos were able to diffuse through the cornea and vitreous humor at a faster rate than unmodified Exos. Modified Exos also resulted in a higher uptake of photoreceptors and increased transfection with encapsulated Enhanced Green Fluorescent Protein (eGFP) mRNA compared to unmodified Exos [158].

Modified Exo-based therapy has the potential to be a viable cell-free therapeutic approach as engineering Exos and their cargo can be modified to target various diseases. Currently, however, Exo therapy has many delivery limitations that hinder its application in ocular surface treatments. Another challenge is that it is difficult to obtain MSC- and iPSC-derived Exos with high purity and yield [159]. Future research will need to focus on the development of efficient technical methods that improve separation efficiency and productivity, as well as address drug delivery efficiency in modified Exos.

#### 5.1.4. Circulating Exos in Biological Fluids as Biomarkers and Messengers

Exos can travel through body fluids and potentially influence physiological conditions such as disease progression and immune responses. Circulating Exos are stable in the bloodstream, as well as in other body fluids. Research efforts have focused on studying circulating Exos since they are actively produced by all cells and are present in circulation, making them a source of cell-specific biomarkers for the diagnosis of different diseases [160,161]. It has also been shown that circulating Exos or EVs are potential sources for liquid biopsies in cancer diagnosis and monitoring, as these biomarkers are abundantly present in various body fluids, such as blood, saliva, tears, sweat, and urine [162,163].

Further, circulating Exos may be used as prognostic biomarkers for retinal diseases, such as diabetic retinopathy, and ocular surface diseases, such as Sjögren’s syndrome, dry eye, and keratoconus. There is considerable interest in identifying tear fluid biomarkers as a non-invasive strategy to detect patients at increased risk of developing diabetic retinopathy. To this effect, differential proteomic profiles in tear fluid from diabetic vs. non-diabetic individuals have been demonstrated [91]. Data analysis identified significant changes in 32 proteins, mostly associated with small EVs and oxidative stress. These candidate biomarkers need to be validated in future clinical studies for use in diagnosis and screening for diabetic retinopathy.

Studies have been conducted to investigate biological fluids to develop more accurate, non-invasive diagnostic techniques. Screening for disease-specific biomarkers is vital for accurate diagnosis and overall improved clinical outcomes. It was found that saliva and tear samples collected from patients with Sjögren’s syndrome exhibited distinct proteomic profiles compared to those from healthy individuals [107]. Interestingly, saliva-derived EVs showed increased content of proteins associated with innate immunity and adipocyte differentiation, whereas tear-derived EVs showed overexpression of proteins involved in TNF-α signaling and B-cell survival. These findings suggest that screening EVs in saliva and tears may increase the diagnostic accuracy of Sjögren’s syndrome and may become a foundation for future research in disease monitoring and the development of new therapeutic strategies.

Among the body fluids studied, tears have emerged as the most favorable source of material for ocular surface disease studies due to their proximity to the cornea and the minimally invasive procedure for collecting samples. Recent research has documented the presence of specific EVs in tear samples from patients with keratoconus. Tear samples from 10 healthy (5 males and 5 females) and 9 patients with keratoconus (4 males and 5 females) were analyzed using the ExoView R100 platform, which allows single phenotypic analyses of EVs [109,152]. The results showed a significant decrease in CD63+/CD9+ and CD63+/CD81+/CD9+ expression in male EVs but not in female EVs with keratoconus. It is of interest that while no significant differences were observed in the total number of tear EVs from healthy and keratoconus patients, significant differences were identified between the sexes [152]. The highlight of the study is also a distinct EV phenotype in tear samples from keratoconus patients compared to healthy individuals. Additionally, tear-derived Exos may have a role in the spread of herpes simplex virus type 1 (HSV-1) in recurrent herpes simplex keratitis (HSK) [164]. Isolated and labeled Exos from the tears of 59 individuals were taken up in a short time by human corneal epithelial cells (HCECs). Notably, following cellular uptake, HSK-associated biomarkers were detected in the infected HCECs by Western blot analysis [164].

Overall, whereas the connection between Exos circulating in bodily fluids and EVs present in the cornea remains to be elucidated, these findings support the potential of circulating EVs as biomarkers for diagnostic and therapeutic applications. Future systematic studies should be conducted to further validate these biomarkers for potential use in disease monitoring and therapeutic applications.

### 5.2. Exosomes as Treatment Targets—Inhibition of Exo Biogenesis or Secretion

EVs such as Exos are involved in many pathological conditions and disease development [165]. Researchers have developed strategies to block the release of subpopulations of EVs or all EVs as a potential research tool to study therapeutic approaches. Identifying and inhibiting EV subpopulations associated with corneal diseases could provide important insights into clinical therapies for these pathological conditions. Studies have evaluated chemical compounds that are capable of blocking or limiting EV formation and release. These drugs can be classified according to their mechanisms of action towards EV biogenesis, which includes ESCRT-dependent and independent pathways, as well as lipid metabolism [166]. Some of the most evaluated drugs are GW4869, Y27632, Calpeptin, and Manumycin A [167,168,169,170]. Each drug has its own corresponding biological target that can inhibit or limit EV biogenesis. GW4869 acts as an inhibitor of membrane neutral sphingomyelinase (nSMase), which is an enzyme that generates biologically active lipid ceramide by the hydrolysis of the membrane lipid sphingomyelin and is involved in Exos generation and release [171,172]. Manumycin A extracted from *Streptomyces parvulus* is pharmacologically classified as an anti-bacterial agent that acts as a selective inhibitor of Ras farnesyltransferases. Due to its involvement in Ras GTPase proteins, manumycin A has recently been identified as an inhibitor of Exos secretion [173,174,175]. GW4869 and manumycin A are inhibitors of Exo generation and release, whereas calpeptin and Y27632 have been shown to be involved in the formation of microvesicles, a subpopulation of EVs [167,176]. For studies focused on the effect of Exos, it is important to utilize the appropriate compound to inhibit Exos to understand its potential as a research tool and how controlling Exos release could serve as a future therapeutic.

As mentioned above, Exo inhibitors could provide a potential approach for disease treatment. They can serve as a valuable research tool by selectively blocking Exo release from cells and potentially offset negative health effects. Research has shown that Exo inhibition can improve response to chemotherapy in small cell lung cancer (SCLC) [177]. A combination treatment of cisplatin/etoposide coupled with a GW4869 Exos inhibitor on small cell lung cancer cell lines enhanced the inhibitory effects of chemotherapy drugs by reducing cell proliferation and inducing apoptosis in these cancer cells [177]. This synergistic relationship between Exo inhibitors and chemotherapy could provide additional opportunities for treating cancers such as SCLC. Several studies have also shown that the inhibition of Exo production using GW4869 or manumycin promotes the wound healing of renal tubular cells, suggesting that the released Exos hinder the rate of wound healing in these cells [174]. Additionally, Exos from corneal stromal cells in keratoconus patients exhibit an altered molecular composition that influences cellular behavior and may contribute to the pathogenesis of the disease [152,178]. Potentially, the inhibition of these Exos could exert a normalizing effect on diseased corneal cells.

The effects of Exo inhibition on corneal diseases have yet to be elucidated. Future in vitro and in vivo studies are needed to further investigate the activities of these drugs and how they behave in corneal cells for potential clinical applications towards corneal pathological conditions.

## 6. Challenges and Opportunities of Exosomes’ Clinical Translation

As mentioned before, Exos exhibit significant therapeutic potential across various biomedical applications due to their intrinsic roles in cellular communication, cargo delivery capabilities, and biocompatibility. Studies regarding the effect of Exos’ treatments in cornea using different model systems have been summarized in Table 1. Despite these advantages, several challenges hinder their widespread clinical application. A fundamental limitation is the heterogeneity of Exo populations, which varies based on the source cell type, culture conditions, and isolation methods, complicating the definition of critical quality attributes (CQAs), such as size, purity, and molecular composition, for clinical translation. Gandham et al. have outlined strategies for standardizing scalable isolation methods, such as tangential flow filtration (TFF) and size-exclusion chromatography (SEC), which offer high recovery rates and minimal loss of biological activity. Advanced characterization tools, including nanoflow cytometry (nano-FCM) and multi-omics profiling, such as liquid chromatography–tandem mass spectrometry (LC-MS/MS), and RNA sequencing (RNA-seq), along with harmonized protocols for sample collection, handling, and storage, are essential for guiding future research and clinical applications [179].

Another major barrier to using Exos clinically is developing effective and sustained drug delivery to ocular tissues. Although the ocular surface offers a convenient route for treating anterior segment diseases due to its accessibility and potential for localized therapy, its utility is hindered by several physiological and anatomical obstacles. These include the multilayered structure of the cornea with poor permeability and dynamic factors, like blinking, tear turnover, and nasolacrimal drainage, all of which limit drug retention and bioavailability. Alternative pathways, like the conjunctival and scleral routes, also face challenges, including high vascularization that promotes systemic absorption and rapid drug clearance. Furthermore, anatomical features, like Descemet’s membrane and the debated posterior Dua’s layer, may affect EV transport across the whole cornea [180]. The physicochemical characteristics of drugs, including molecular size, solubility, and lipophilicity, further complicate targeted and sustained ocular delivery. Table 2 compares the challenges of Exo-based delivery with those of traditional ophthalmic drug delivery methods. Exo-based drug delivery systems have emerged as a promising strategy to overcome several limitations associated with the dynamic environment and multiple physiological barriers of the ocular surface due to their inherent advantages as naturally secreted vesicles by cells. Unlike synthetic carriers, endogenously derived Exos offer high biocompatibility and low immunogenicity and minimize the risk of immune reactions [16,181]. Their nanoscale size and compatible membrane composition facilitate penetration into target cells, including the corneal epithelium and conjunctiva [182]. Engineered Exos with specific surface ligands or integrins can bind selectively to target cells or tissues, enabling the sustained release of therapeutic cargo [183]. Additionally, the Exos’ cargos, such as miRNAs and proteins, are protected from degradation by their lipid bilayer encapsulation [184,185]. As previously noted, their quick removal from the eye surface, caused by tear film turnover and regular blinking, reflects the limitations of other topical delivery agents and significantly decreases bioavailability [183]. Another important concern is the low efficiency of drug loading. Although passive loading methods are straightforward, they frequently lead to poor encapsulation. Recent advances in therapeutic nanoparticles, including Exo-based systems, also emphasize the need for delivery approaches that preserve bioactivity while ensuring safety and distribution, highlighting this translational challenge [186]. To improve drug incorporation, various active techniques have been developed, including electroporation, sonication, extrusion, freeze–thaw cycles, and chemical transfection; however, some of these methods carry the risk of damaging the Exo structure [54,187,188]. Hybrid constructs combining Exos with liposomes are also being explored to increase stability and payload capacity [85].

In conclusion, Exo-based delivery systems offer a compelling approach to overcoming many of the conventional limitations in ocular surface drug delivery. Their future clinical success depends on addressing current challenges related to stability, scalability, cargo loading, tissue penetration, and regulatory standardization. Importantly, future research should also focus on establishing reproducible dosing metrics for Exo-based ocular therapies to ensure therapeutic consistency, safety, and efficacy across diverse patient populations. Continued optimization of delivery strategies and mechanistic insights will be essential to fully realize their therapeutic potential in treating ocular surface diseases.

## 7. Discussion and Perspectives

**Figure 3:**
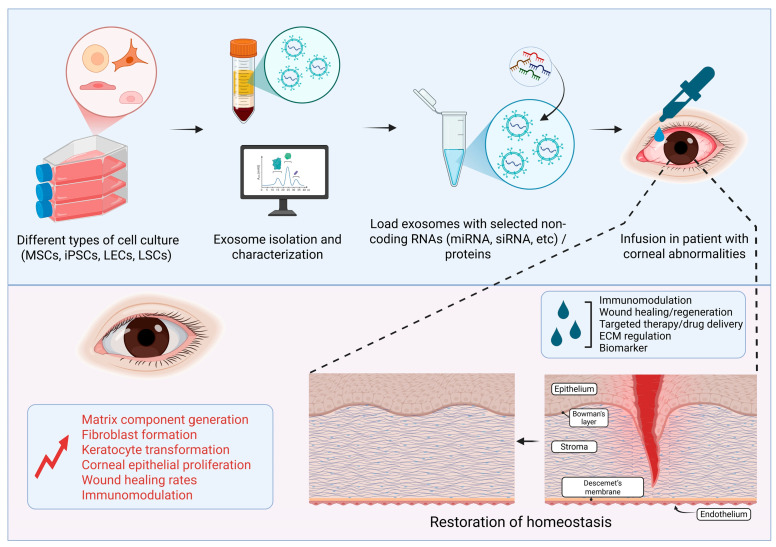
Schematic showing the therapeutic potential of Exos derived from eye fluids or mesenchymal stem cells (MSCs), induced pluripotent stem cells (iPSCs), limbal epithelial cells (LECs), and limbal stromal cells (LSCs) in corneal repair and regeneration (Image created in BioRender).

Exos have emerged as a promising therapeutic modality in ophthalmology, particularly for corneal diseases, owing to their unique biological properties, including the ability to mediate intercellular communication, deliver bioactive molecules, and modulate cellular functions. Advances in isolation and engineering technologies have further underscored their dual potential: as biomarkers for diagnostic applications and as therapeutic vehicles capable of delivering specific molecular cargos directly to target cells. As of 2023, 288 clinical trials involving Exos and 127 trials focused on EVs were registered on ClinicalTrials.gov [189]. Among the most compelling features of Exos is their cargo of miRNAs, which play critical roles in regulating key cellular processes, such as corneal homeostasis, wound healing, and inflammatory responses. Exosomal miRNAs are particularly attractive as biomarkers due to their intrinsic stability, resistance to enzymatic degradation, and detectability in bodily fluids. These characteristics position them as valuable tools for early diagnosis, prognosis, and therapeutic monitoring of corneal disorders. Notably, studies have revealed differential miRNA expression profiles in Exos derived from diabetic versus non-diabetic corneal cells, correlating with variations in wound healing capacity. Additionally, other studies investigated the downregulation of genes associated with fibrotic markers, inflammation, and tissue damage in fibrosis and dry eye disease, highlighting the mitigating effects mediated by specific miRNAs. These findings emphasize the potential of exosomal miRNAs to advance personalized medicine in ocular surface disease management. Engineered Exo-based therapies represent a novel and expanding frontier in regenerative ophthalmology. Genetic and molecular modifications can enhance the specificity and efficacy of Exos for targeted therapy. As illustrated in Figure 3, one potential strategy involves deriving Exos from distinct cell sources and loading them with tailored non-coding RNAs to improve therapeutic precision. Moreover, surface modifications, such as polyethylene glycol (PEG)ylation or the incorporation of cationic motifs, have been shown to significantly enhance Exos’ bioavailability and targeting capabilities, thereby improving delivery across barriers such as the corneal epithelium. Another emerging approach involves pharmacologically inhibiting the release of pathological Exos, potentially modulating disease progression or enabling controlled tissue regeneration. Nonetheless, the specificity, safety, and risk of off-target effects associated with such inhibitors require extensive investigations prior to clinical translation. Despite encouraging preclinical findings, several challenges hinder the clinical application of Exo-based therapies. These include the lack of standardized protocols for Exo isolation, characterization, and large-scale production. Addressing these knowledge gaps through in-depth mechanistic studies will be crucial for advancing the field. Regulatory barriers also pose significant hurdles, as no Exo-based therapies have yet received approval from regulatory agencies. Key concerns surrounding Exo-based therapies include the possibility of immune reactions, risks of contamination, such as co-isolated non-exosomal particles, residual media components, or microbial endotoxins, and the potential for adverse effects, including unintended oncogenic transformations. In conclusion, Exos and their miRNA cargos offer substantial promise as innovative diagnostic and therapeutic tools in ophthalmology, with particular relevance to corneal disease. Future research should prioritize the refinement of isolation techniques, the development of scalable production platforms, the elucidation of cargo sorting mechanisms, and the optimization of delivery systems. Overcoming these challenges will be essential to enabling the successful clinical translation of Exo-based therapies and advancing personalized approaches in ophthalmic care.

**Table 2:** Comparison of exosomes with conventional ophthalmic drug delivery bottlenecks.

Challenge	Conventional Delivery (e.g., Eye Drop)	Exosome-Based Delivery
Bioavailability	Rapid tear clearance, <5% [190]	Slightly improved but still not cleared unless modified [191]
Penetration	Poor corneal and conjunctival penetration [192]	Enhanced via vesicle fusion and receptor-mediated uptake [182]
Target Specificity	Largely non-specific	Surface modification allows targeted delivery [183]
Stability of Therapeutics	Susceptible to degradation [193]	Cargo protected within lipid bilayer [184]
Manufacturing	Simple and scalable	Technically demanding, not yet standardized [194]
Regulatory Pathway	Well-defined	Still evolving and undefined [189]

## 8. Conclusions

Exos have emerged as transformative agents in the field of biomedical and translational research, particularly in corneal care and disease. They can be easily delivered and monitored in vivo and serve a dual role as both diagnostic biomarkers and therapeutic agents, posing as both tools and targets. Their cargos, stability, and cell-targeting abilities highlight their potential for precise diagnostics and personalized treatments. While advances in bioengineering and delivery strategies have driven the field forward, clinical translation remains limited by technical, regulatory, and safety challenges. Future interdisciplinary research focusing on standardization, scalability, and mechanistic insights will be pivotal in reaching the full potential of Exo-based therapies in regenerative ophthalmology.

## Figures and Tables

**Table 1:** A summary of studies regarding the effects of exosome treatments.

Origin of Exos	Model	Results
Corneal epithelial cells (CECs)	Mouse in vitro	Increased fibroblast and human umbilical vein endothelial cell proliferation [17] Transformed keratocytes to myofibroblasts [17]
Limbal epithelial cells (LECs)	Human in vitro	Induced wound closure and proliferation in primary LSCs [96]
Limbal stromal cells (LSCs)	Human in vitro	Induced wound closure in primary LECs and organ-cultured corneas [18]
Corneal stromal MSCs	Human in vitro	Induced wound closure and proliferation in human CECs [95]
	Mouse in vitro	Induced wound closure and proliferation in mouse cornea [95]
Human CECs, corneal fibroblasts, and endothelial cells	Human in vitro	Induced wound closure and proliferation in human CECs [97]
	Human in vitro	Enhanced proliferation and migration of primary human LECs in dry eye disease model (high osmotic stress) [99]
	Mouse in vitro	Enhanced tear production in dry eye mouse model
Bone marrow-derived MSCs	Human in vitro	Induced wound closure and proliferation in human CECs [100]
	Mouse in vivo	Enhanced wound healing of mice with corneal injury [100]
Adipose-derived MSCs	Rabbit in vitro	Enhanced wound healing in rabbit CECs [125]
Mouse amniotic fluid MSCs	Mouse—in vitro	Enhanced wound healing in mouse cryoinjured cornea [126]
Human umbilical cord MSCs	Rat—in vitro	Subconjunctival injection promoted the healing of rat corneal damage [131]
	Human—in vitro	Increased proliferation and migration of human CECs [131]
Embryonic stem cell-derived MSCs	Rat—in vitro	Enhanced corneal healing and reduced corneal scarring caused by irregular phototherapeutic keratectomy in rats [136]
	Human—in vitro	Human corneal epithelial cells [136]
Human bone marrow-derived MSCs	Human—in vitro	Decreased ER stress-related genes and apoptosis in human corneal endothelial cells [138]
Corneal stromal stem cells	Mouse—in vitro	Normal tissue morphology and prevention of scarring in mouse damaged corneas [138]

ER, endoplasmic reticulum; LSCs, limbal stromal cells; MSCs, mesenchymal stem cells; CECs, corneal epithelial cells.

## Data Availability

Not applicable.
